# Reference-free NOE NMR analysis†

**DOI:** 10.1039/d0sc02970j

**Published:** 2020-08-31

**Authors:** Martin R. M. Koos, Karl H. G. Schulz, Roberto R. Gil

**Affiliations:** Department of Chemistry, Carnegie Mellon University 4400 Fifth Avenue Pittsburgh Pennsylvania 15213 USA rgil@andrew.cmu.edu

## Abstract

Nuclear Overhauser Effect (NOE) methods in NMR are an important tool for 3D structural analysis of small molecules. Quantitative NOE methods conventionally rely on reference distances, known distances that have to be spectrally separated and are not always available. Here we present a new method for evaluation and 3D structure selection that does not require a reference distance, instead utilizing structures optimized by molecular mechanics, enabling NOE evaluation even on molecules without suitable reference groups.

## Abstract 2

A quantitative Nuclear Overhauser Effect (NOE) analysis approach that avoids the use of and internal reference distance to perform molecular configuration selection.

## Graphical abstract

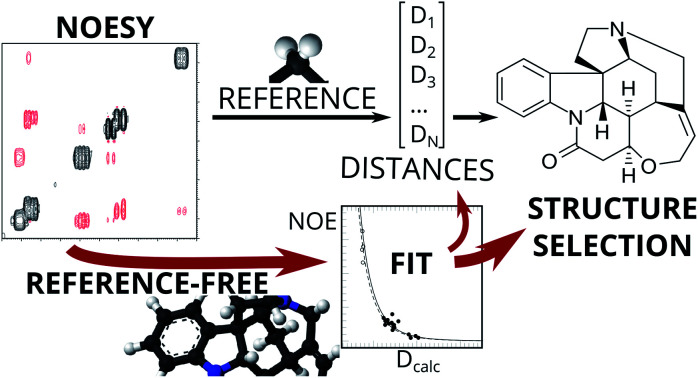

## Introduction

The nuclear Overhauser effect or enhancement (NOE) is one of the most extensively and intensively used NMR parameters to determine molecular 3D structure. In contrast to chemical shift and *J*-coupling, which are effects of the electron shell, NOE is a relaxation process caused by a direct dipole–dipole interaction between nuclei.

In 1953, Overhauser predicted that microwave irradiation on electron transitions in certain metals would transfer magnetization from electrons to nuclei, enhancing nuclear spin polarization a thousandfold.^1^ This was experimentally demonstrated in the same year by Carver and Slichter,^2,3^ and the concept opened up the field of dynamic nuclear polarization (DNP).^2,3^ An analogous enhancement between two nuclei, the nuclear Overhauser effect was first observed by Solomon in 1955 and was explained as a so-called cross relaxation between nuclear spins in proximity.^4,5^ From there, it took ten years for NOE to become appreciated as an analytical tool applied qualitatively to assignment and structure selection problems.^6–8^ The quantitative interpretation of NOE intensities for the use of measuring internuclear distances followed swiftly.^9–11^

While initially frequency selective 1D experiments had to be performed for each proton of interest, the two-dimensional NOE spectroscopy (NOESY) experiment provided a way to acquire complete NOE data in a single acquisition run.^12,13^ The last gap in NOE applicability was medium-sized molecules, that have inherently weak NOE if molecular tumbling rates are close to the Larmor frequency, which was closed by the rotating frame Overhauser spectroscopy (ROESY) experiment (the creative name “cross-relaxation appropriate for minimolecules emulated by locked spins”, CAMELSPIN, given by its inventors did not catch on).^14,15^

Treating a two-proton system quantitatively, the integrated cross-peak volume in a 2D NOESY has an *r*^−6^ dependence on the spatial separation between the nuclei.^9,10^ In systems featuring more spins, relayed transfer can be observed.^16^ This aspect of NOE, called spin diffusion, dominates relaxation in large molecules or at high Larmor frequencies, where it limits experimental parameters and complicates evaluation.^13,17^ While spin diffusion is usually negligible in small molecules, NOE was used strictly in a semi-quantitative way for a long time due to other relaxation effects. This changed when Hu and Krishnamurthy revisited an old technique of peak normalization that they called “peak amplitude normalization for improved cross-relaxation correction” (PANIC).^18–21^ It was elaborated extensively by Butts's research group, opening a new chapter of accurate and precise NOE-derived distances.^22,23^

Integrated NOESY peak volumes after PANIC are

1 *a*_AB_/*a*_AA_ = tanh(*b* × *r*_AB_^−6^) ≈ *b* × *r*_AB_^−6^

where *a*_AB_ corresponds to the integrated cross-peak volume, *a*_AA_ to the diagonal volume, and *b* is a proportionality factor.^18–20^ The parameters making up *b* are known and extensively discussed in the literature.^11,13^ In essence, the prediction of *b* is complicated because it depends on the local correlation time *τ*_C_ which depends on global and local dynamics. As such, gross approximations are typically used. Assuming a rigid molecule with a single correlation time *τ*_C_ for all interatomic vectors, there is a single constant *b* for the whole molecule. Choosing a suitable known interatomic reference distance *r*_ref_ and the corresponding reference intensity *a*_ref_, eqn (2) transforms all NOE integrals *a*_i_ into interatomic distances *r*_i_ without calculation of *b* from other measurements or simulations.^9,23^

2 *r*_i_^−6^ = *a*_i_ × *r*_ref_^−6^/*a*_ref_

Commonly, geminal protons in CH_2_ groups or vicinal protons on double bonds or aromatic systems are used, since their distance varies little with conformational changes and typical distances are tabulated.^24^ Looking at prominent recent works on NOE, the concept of an internal reference for absolute NOE distance measurement was applied with great success by the small molecule community.^23,25,26^ For molecules without suitable internal distance reference or overlapping reference signals of insufficient chemical shifts separation, such analysis is still deemed impossible and not attempted.

To improve the accuracy of the reference distance, the structure in question is nowadays often generated *in silico* and optimized by molecular mechanics or DFT. In this process, it became apparent that in many cases the reference atoms at close distance show different relaxation behavior than the other, more distant, atom pairs in the rest of the molecule. An elegant approach to improve the clarity of structure selection is therefore to adjust the reference distance to better match the whole molecule.^27,28^ While the concept of such a reference was never abandoned, we think this approach holds the key to NOE analysis without that reference, as will be shown below.

## Results and discussion

We propose that the proportionality factor *b* can be determined on the fly from a simple fitting procedure using the distances from the (*in silico* generated) molecular structure and the experimental NOE data by minimizing a quality criterion like the sum of squared errors,3
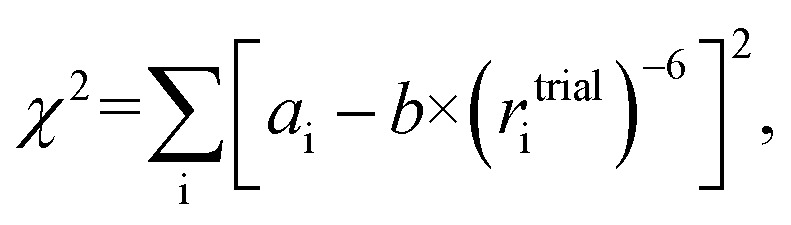
for the whole molecule, without the need of an internal reference.

**Scheme 1:**
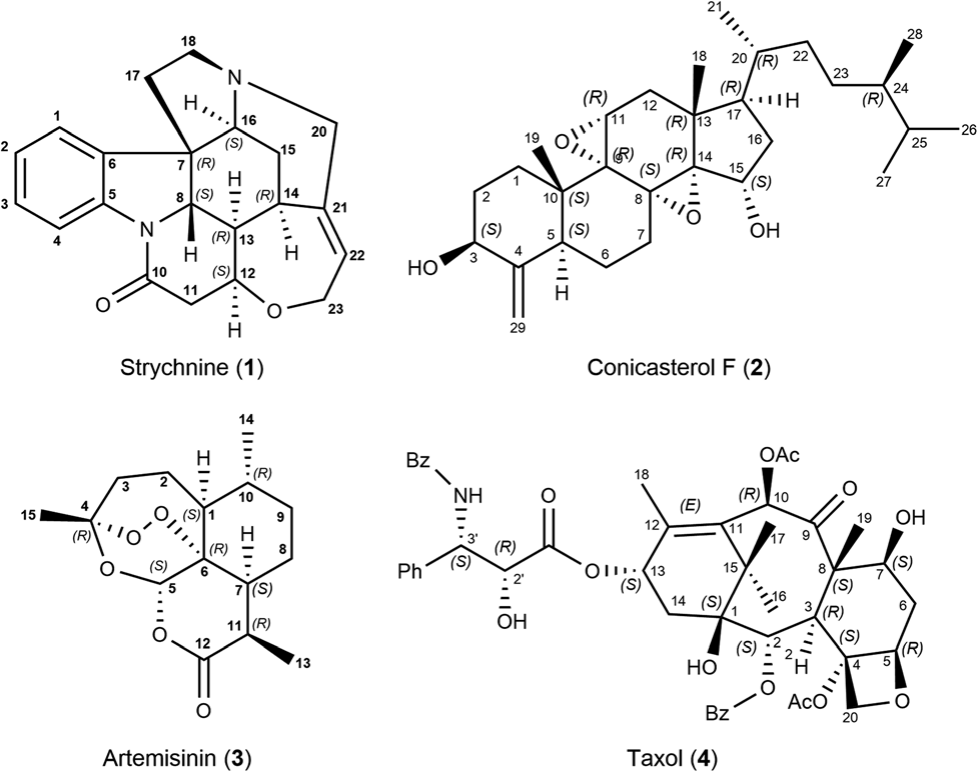
Strychnine (**1**), the correct configuration is labelled *RSSRRS* in this work. Conicasterol F (**2**), the correct configuration is the 8,14-α-epoxide as shown. Artemisinin (**3**), the correct configuration is labelled *RSRSRR*. Taxol (**4**) configuration selection was focused on the taxane skeleton, not on the highly flexible side chain.

To showcase the new method, a 2D-NOESY experiment with 500 ms mixing time was acquired on strychnine (**1**) in CDCl_3_.^29^ A total of 21 integrated intensities were obtained and normalized with respect to their diagonal signal intensity.^21,30^ Among the peaks are 4 intra-CH_2_ signals that could conventionally be used as reference and 17 cross-peaks between more distant protons that hold structural information. Molecular mechanics-generated structures (using MMFFs) of strychnine and 12 energetically feasible diastereoisomers were used for comparison and structure selection (see the ESI† for more details). Each isomer is labeled using the stereochemical descriptors *R*/*S* for each chiral carbon in the order C-7, C-8, C-12, C-13, C-14 and C-16. For example, the correct absolute configuration of strychnine is 7*R*,8*S*,12*S*,13*R*,14*R*,16*S*, shown in Scheme 1, hence it is labeled as *RSSRRS*. The same labelling scheme is used for all molecules in this study.

**Fig. 1:**
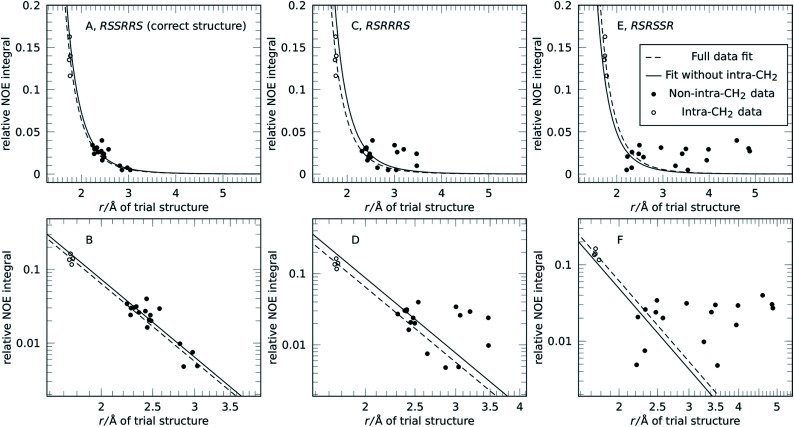
Fit of trial structure distance to experimental NOE intensity for three different structures related to **1**: (A and B) *RSSRRS*, the correct configuration of strychnine (**1**), (C and D) *RSRRRS*, an epimer of strychnine, and (E and F) *RSRSSR* in linear (A, C and E) and logarithmic plot (B, D and F). Using all available NOE data (○ and ●) for evaluation, the dashed line (– –) is obtained. Without intra-CH_2_ data (● only), the solid line (—) is obtained. In all cases the fit result can replace a reference integral calibration for distance measurement.

The concept of reference-free NOE fitting is displayed in Fig. 1. No data point is explicitly defined as reference, instead, for each trial structure, all data is combined and the error minimized according to eqn (3). The resulting curve (dashed line) represents a model that allows calculation of distances from intensities if desired, like a conventional reference would. For clarity and space reasons, the fitting to (A, B) the correct configuration of strychnine, *RSSRRS*, (C, D) its epimer at C-12, *RSRRRS*, and (E, F) isomer *RSRSSR*, corresponding to best fitting, second best fitting, and poor fitting quality, respectively, are displayed. Fig. S1 of the ESI† shows the fitting for all diastereoisomers of the ensemble.

The data displayed in Fig. 1 shows that the distances between geminal protons, *i.e.* intra-CH_2_ distances, hardly change with configuration and conformation, which is why they are conventionally used as references. Their integrated intensities are about one order of magnitude higher than all other intensities and show only small variation. All other NOE intensities and distances vary strongly and this is where the structural discriminatory power lies.

Due to their large intensity, intra-CH_2_ data dominate this fit but depending on molecule and spectral quality, they may not be available. To resemble such a molecule without usable CH_2_ intensities altogether, these data points were excluded in the next step and the reference-free fit was performed on this reduced dataset (filled black points in Fig. 1). The resulting curves (solid lines) are more spread for the different structures but the discriminatory power is retained as will be shown below. Fig. 1 also shows that for correct (but also incorrect) structures, the curves obtained from the fitting procedure lie within the expected range of conventional references. This enables absolute interpretation of NOE integrals, *i.e.* distance measurement, for all kinds of molecules, even those without appropriate reference proton pairs.

Different from strychnine, the next three examples contain methyl groups, which undergo internal rotations. These motions require averaging of proton–proton distances, depending on rotation rates compared to molecular tumbling. Even in small molecules methyl group rotation is fast compared to molecular tumbling and distances should be averaged as 〈*r*^−3^〉 instead of 〈*r*^−6^〉, as nicely described in the seminal article by Koning, Boelens, and Kaptein in 1992.^31^

**Fig. 2:**
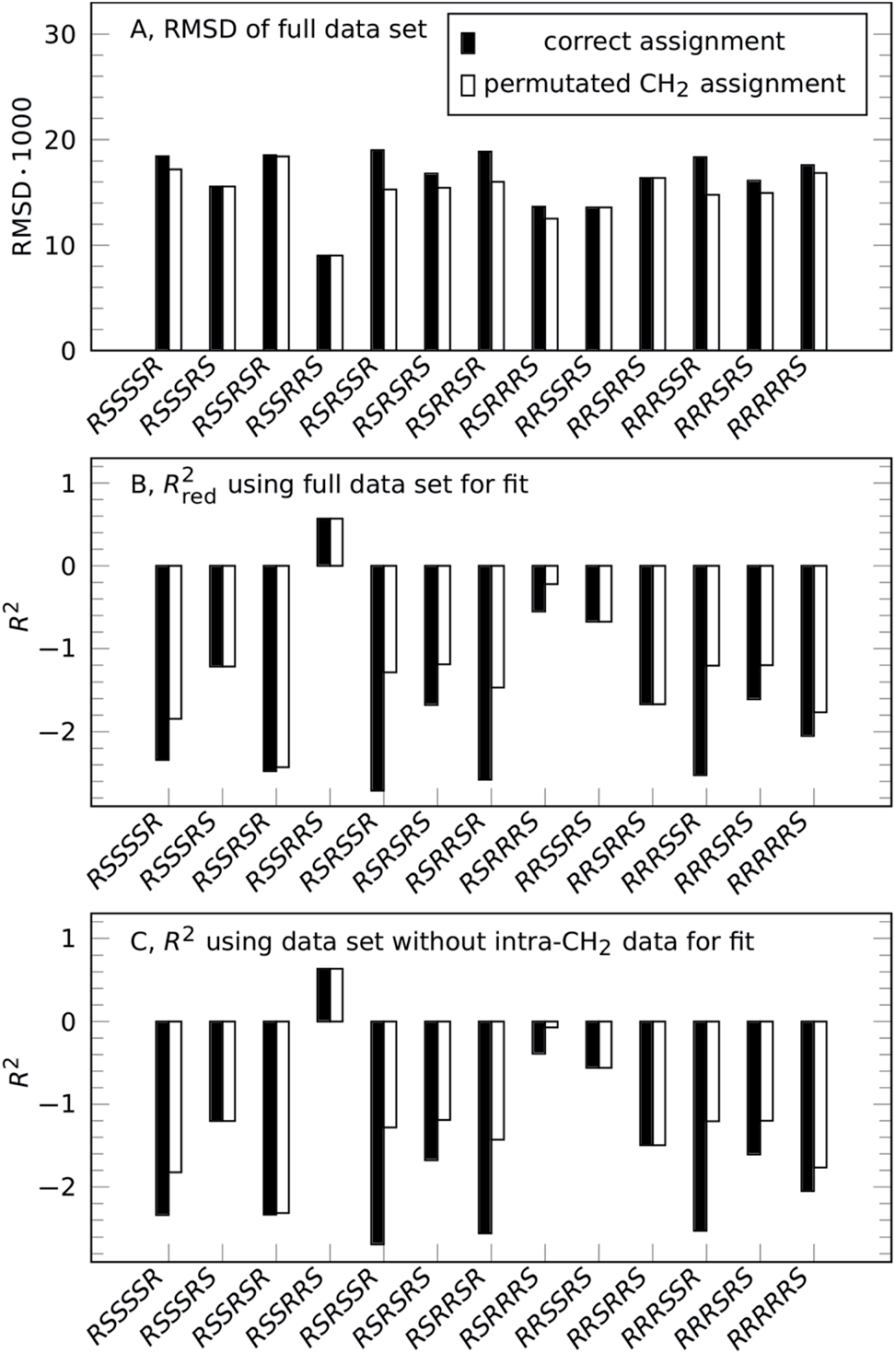
Result of reference-free NOE-based structure selection of **1**, either for the correct assignment (solid black bars) or for individually optimized CH_2_ assignment (unfilled bars). The correct configuration of strychnine is *RSSRRS*. (A) RMSD of fitting structure distances to experimental NOE integrals using the full set of NOE intensities (lower value indicates better fit). (B) *R*_red_^2^ analysis of the same fit as (A) using a reduced dataset excluding geminal NOE intensities (higher positive value indicates better fit). (C) *R*^2^ of a fit to reduced dataset excluding geminal NOE intensities.

Going from the reference-free fitting procedure to structure selection, Fig. 2 shows the result of trial structures evaluated using different scoring functions, RMSD or *χ*^2^ and an *R*^2^-like score. The fourth configuration, *RSSRRS*, corresponds to the correct structure, as confirmed by all scoring functions. While RMSD identifies the correct structure (Fig. 2A), it is not always the best measure of model performance. A good way to convey agreement between data and model is *R*^2^, the so-called coefficient of determination. It is reflecting how large the spread of data is and how well it follows the model. Data that poorly follow the model can show negative *R*^2^ values for non-linear functions, like in the current method. Since intra-CH_2_ distances have almost no discriminatory power but would dominate *R*^2^, a different score, *R*_red_^2^, is defined as *R*^2^ excluding intra-CH_2_ corresponding data points (but they still contribute to the fit).

As can be seen in Fig. 2B, as with RMSD, the parameter *R*_red_^2^ selects the correct structure (highest value). As a bonus, the difference between correct and incorrect structures is visually well pronounced.

The selection can also be performed for the case without intra-CH_2_ data, as mentioned before, corresponding to filled circles and solid lines in Fig. 1. Fig. 2C shows the results of this truly reference-free selection; since intra-CH_2_ data is excluded completely, *R*_red_^2^ = *R*^2^. The results are almost as clear as in the previous case and again, the correct structure is selected. Inclusion of intra-CH_2_ data can be advantageous in some cases, but as intra-CH_2_ NOE often deviate from the behavior of all other proton interactions, the last scoring method, *R*^2^ without intra-CH_2_ data points, is the most applicable method and will be used in the following examples.

The method presented here requires prior determination of constitution and assignment of all signals. Accordingly, it is shown here for complete assignment of all protons, as is available for strychnine.^30^ Of course, full assignment is not always available – for example, assignment of diastereotopic CH_2_ groups often requires knowledge of relative configuration and conformation.

To show viability even without such knowledge, a permutation approach can be utilized to obtain the full assignment alongside the structure selection.^32,33^ Strychnine contains six CH_2_ groups but the C17-methylene shows insufficient proton chemical shift separation to be used, so there are 32 ways to assign geminal proton pairs. Each trial structure was therefore evaluated for all 32 combinations and the individual best assignments were used. For the correct structure, these scores are unchanged, confirming the assignment. Incorrect structures can reach better scores by alternative assignment, lowering the separation between correct and best incorrect structure. However, as the unfilled bars in Fig. 2 show, the correct structure is still selected by all scoring functions.

The reference-free method was then applied to conicasterol F (**2**), a marine natural product, to reproduce the determination of relative configuration without hydrogen atoms attached to the carbon atoms in question.^34^ In the original study, the molecule was determined by 2D-NMR methods to be either the 8,14-α-epoxide or the 8,14-β-epoxide isomer. Quantitative 1D-ROE data was interpreted using geminal vinylic protons as reference and a subset of key distances that differ significantly between the two isomers were selected. The fit between ROE-derived distances and DFT-calculated structures identified the 8,14-α form as correct configuration of conicasterol F, which was confirmed by ^13^C chemical shift predictions.

**Fig. 3:**
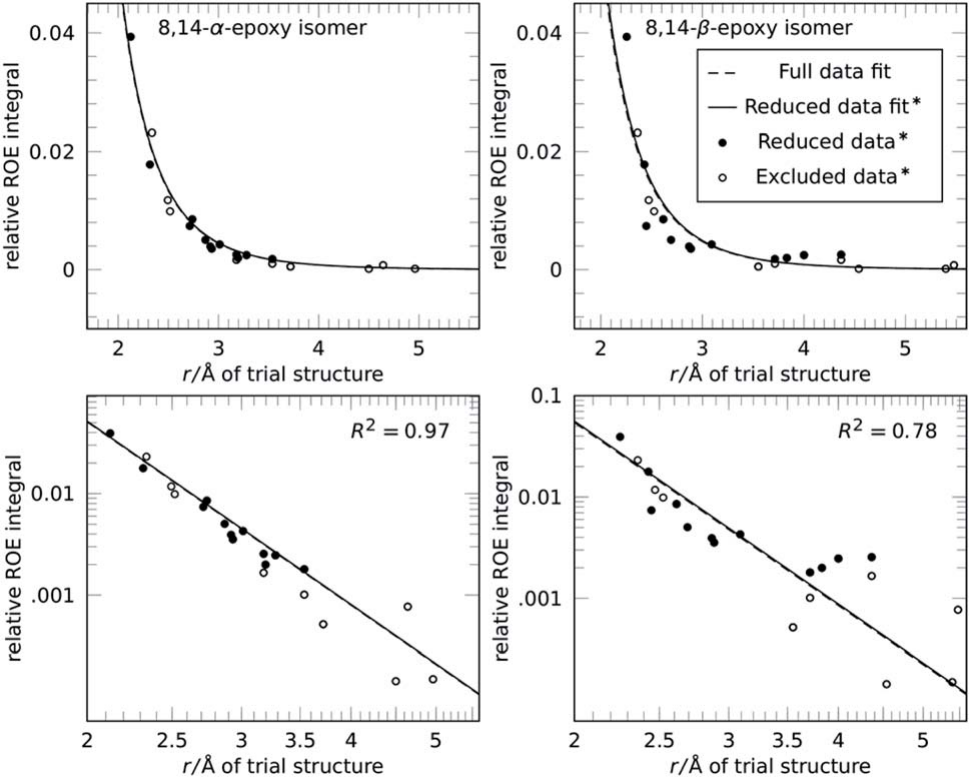
Relative configuration determination of **2**, conicasterol F with 8,14-α configuration (left) and 8,14-β isomer (right) in linear (top) and logarithmic plot (bottom). 1D-ROE data and coordinates were taken from a reference.^34^ The authors analysed the two calculated structures and identified a subset of distances that change between the isomers to improve their selection (● reduced data). In the reference-free selection both the reduced (●) and the full subset (● and ○) show essentially the same fit results and score. Conventional and reference-free analysis select 8,14-α as correct structure.

Using the ROE-data and coordinates provided in the study, the reference-free analysis results are essentially the same for the refined subset of ROE intensities as well as for the unrefined full dataset as can be seen in Fig. 3. The configuration selection is reproduced effortlessly and the 8,14-α-epoxide is selected.

Next we applied the method to the important anti-malaria drug artemisinin (**3**).^35,36^ Artemisinin has 7 stereogenic centers and therefore 64 configurations or 32 pairs of enantiomers. The correct absolute configuration of artemisinin is 1*S*,4*R*,5*S*,6*R*,7*S*,10*R*,11*R* (see Scheme 1) and represented here as *SRSRSRR*. It is more flexible than strychnine (**1**) and several configurations have more than one conformation at energies relevant at room temperature. The generation of stereoisomers was followed by a conformational search with 3 kcal mol^−1^ cutoff energy and removal of redundant conformations (see the ESI† for more details). A total of 12 integrated NOE intensities, including 2 intra-CH_2_ integrals, were obtained from a 2D-NOESY experiment and normalized with respect to their diagonal signal intensity. ^1^H signal assignment was taken from literature.^37^ All conformations then underwent NOE analysis excluding the two intra-CH_2_ datapoints and the highest scoring conformation was used to represent its configuration in the selection.

**Fig. 4:**
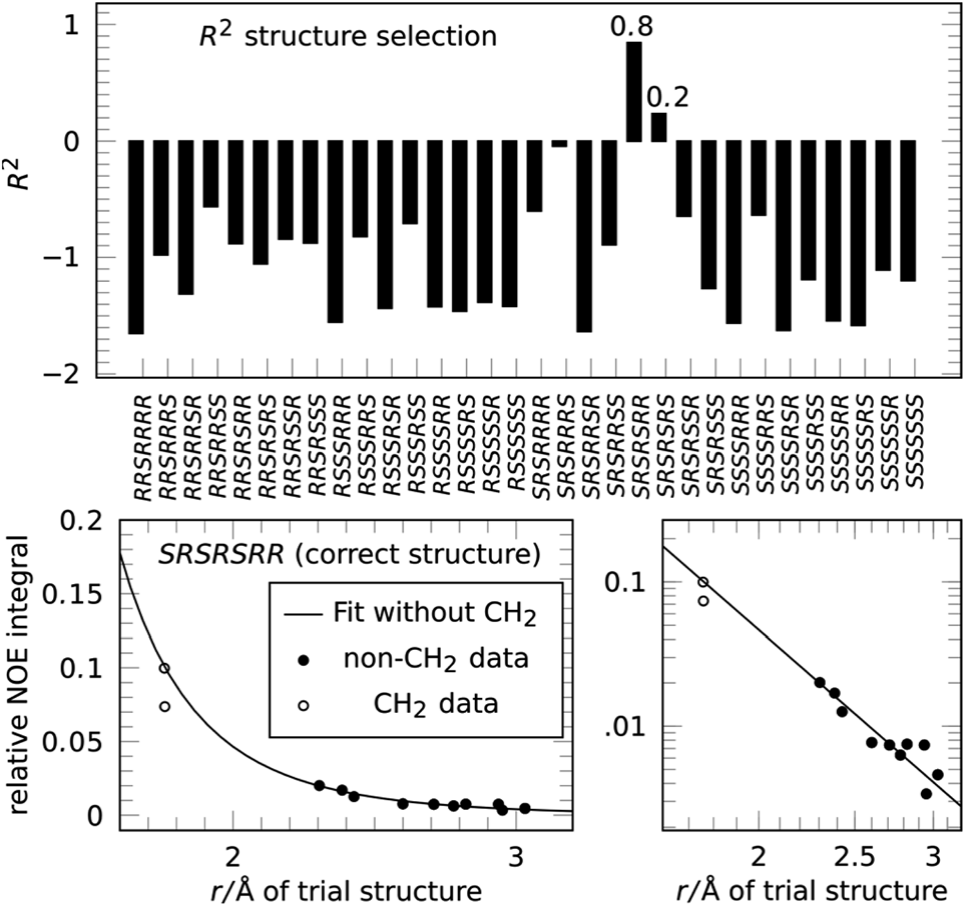
Applying the NOE configuration selection to **3** (top) clearly selects the correct configuration of artemisinin with *R*^2^ = 0.84, the epimer at C-4 only reaches *R*^2^ = 0.24. The fit result (bottom) shows that the reference-free fit of the correct structure agrees well with a conventional reference approach.

The resulting score for all configurations of **3** is shown in Fig. 4. The correct structure of artemisinin is selected with *R*^2^ = 0.84, with a second-best fit for the epimer at C-4 with *R*^2^ = 0.24, all other configurations show negative scores. The fit for the correct structure is also shown in Fig. 4, the curve shows that in this case, the proportionality constant derived from the fitting procedure lies well within the calibration achieved by using one of the two conventional reference signals. This means, distance measurements based on the reference-free method are as accurate as distance measurements based on a conventional reference.

**Fig. 5:**
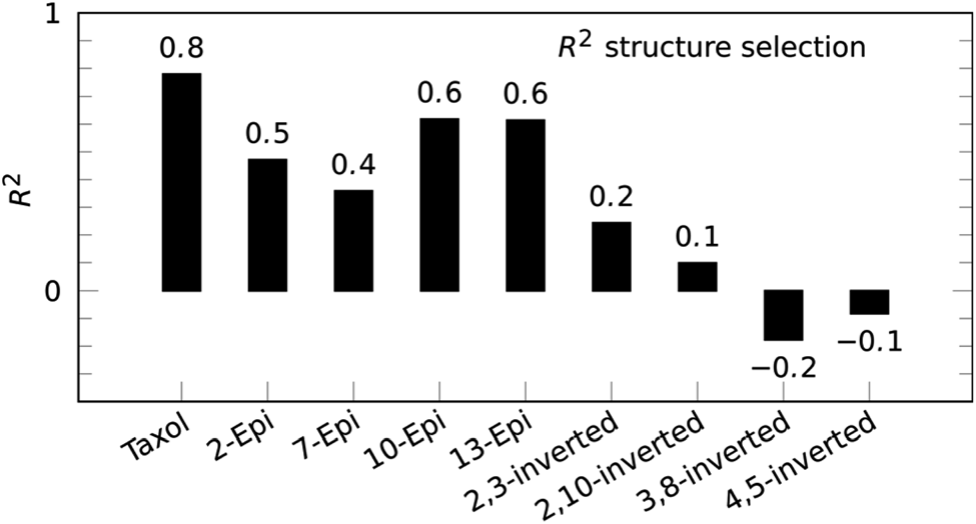
Reference-free taxol (**4**) structure selection selects the correct configuration out of a set of stereoisomers with inverted stereocenters of the taxane skeleton.

Paclitaxel (**4**) was chosen as a fourth example, an important chemotherapy medication sold under the brand name taxol among others and historically referred to by that name.^38–40^ Taxol has a total of 11 stereocenters, corresponding to 1024 pairs of enantiomers, and most configurations have a large conformational space of more than 10 conformations, due to the high flexibility of the side-chain at C-13. Analyzing all possible structures, as well as performing a conformational analysis, is beyond the scope of this publication. Instead, a set of 8 diastereoisomers derived from the correct configuration were generated by inverting key stereocenters of the taxane skeleton. This was followed by a conformational search with 3 kcal mol^−1^ cutoff energy and removal of redundant conformations (see ESI† for more details). ^1^H signal assignment was taken from literature.^41^ For this example, the application of the reference-free NOE method was focused on the selection of configuration of the taxane skeleton, leaving the NOEs involving the hydrogens of the side-chain out of the fitting procedure. Using a total of 53 NOE enhancements, all conformations underwent the reference-free NOE analysis to be scored and the highest conformer score was used to represent each configuration. The result of the selection is shown in Fig. 5, and again, the correct configuration is selected.

## Conclusions

NOE is a very interesting and unique method for 3D analysis of molecules. In spite of well-known problems concerning conformational exchange and internal motions like methyl group rotation, and the possibility of additional, indirect, transfer pathways (spin diffusion), the simple two-spin approximation, assuming intensity proportional to *r*^−6^, has been used successfully for a long time for small molecules.

The internal reference distance that is almost always used for calibration, however, is nothing but a global scaling factor of distances for the whole molecule and might not even match the distances it is supposed to measure. When computer generated structures are available, instead, a fit can be performed to provide a calibration for distance measurement. For structure selection within a limited pool of configurations or conformations, this demonstrably does not limit the discriminatory power. Of course, the method requires a suitable pool of trial structures and can provide false positive results if the correct structure is not included.

Since this is a fitting procedure similar to the way RDCs and RCSAs are analyzed, it is aimed at structure selection and not at a *de novo* determination of 3D structure from molecular constitution. If the correct configuration is known, however, the method is also a powerful tool to calculate absolute distances even in absence of a calibration reference. The analysis is not limited to NOESY spectra and can similarly be applied to 1D-NOE data or rotating frame Overhauser experiments (ROE/ROESY).

For a rigid molecule like strychnine, a single conformation is sufficient for selection.

Multi-conformational averaging can be a straightforward extension if the populations are known, for example from energy levels and Boltzmann distribution.^42^ Similarly, conformation analysis can be achieved by varying populations to match experimental data.^43^ We expect a single fit for all conformations, corresponding to a single correlation time *τ*_C_, to be sufficiently accurate for configuration selection as long as the exchange rate is faster than the relaxation rates.^24^

The method shows a new perspective of NOE analysis and structure selection that is reference-free and therefore can be applied to all molecules, even those without appropriate geminal or vicinal aromatic (or *cis* double bond) proton pairs at all. For these molecules, NOE data can now be used for computer-assisted configuration selection in a multi NMR parameter protocol alongside residual dipolar couplings (RDCs), residual chemical shift anisotropy (RCSAs), chemical shifts, and *J*-couplings.^37,44,45^ It is an excellent choice to be applied in fields such as peptide and carbohydrate research, organic synthesis, natural products, and medicinal chemistry.

## Conflicts of interest

There are no conflicts to declare.

## Supplementary Material

SC-011-D0SC02970J-s001

SC-011-D0SC02970J-s002

SC-011-D0SC02970J-s003

SC-011-D0SC02970J-s004

SC-011-D0SC02970J-s005

SC-011-D0SC02970J-s006
