# Supplementary material for: Reference-free NOE NMR analysis
Source: Chem Sci. 2020 Aug 31;11(36):9930–6. doi: 10.1039/d0sc02970j (PMC8162118; doi:10.1039/d0sc02970j)
Supplement: SC-011-D0SC02970J-s001 [file SC-011-D0SC02970J-s001.pdf]

Electronic Supplementary Material (ESI) for Chemical Science.
This journal is © The Royal Society of Chemistry 2020

Reference-free NOE NMR analysis: Supporting Information

Martin R. M. Koos, Karl H. G. Schulz, Roberto R. Gil

Department of Chemistry, Carnegie Mellon University, 4400 Fifth Avenue, Pittsburgh, Pennsylvania 15213, United States.

Contents

NMR parameters for NOESY 1
Structure generation 1
Note on Matlab source code 2
Fit results for strychnine configuration selection 3
Additional quantitative strychnine scoring data 8
Fit results for artemisinin configuration selection 10
Fit results for taxol configuration selection 16

NMR parameters for NOESY

The NOESY spectra of strychnine (**1**) and artemisinin (**3**) were acquired at 300 K on a Bruker AVIII NMR spectrometer (operating at 500.13 MHz for ^{1}H) equipped with a room temperature BBFO SMART probe head, while the NOESY spectrum for paclitaxel (taxol) (**4**) was acquired at 300K on a Bruker NEO 500 NMR spectrometer (operating a 500.00 MHz for ^{1}H) equipped with the multinuclear BBO Prodigy cryoprobe.
The pulse program noesygpphzs (phase-sensitive gradient-enhanced NOESY with Thrippleton-Keeler-type z-Filter element) of a Bruker TopSpin installation (version 3.5pl7 for AVIII and version 4.03 for NEO) was used to record all the 2D-NOESY spectra. For **1** and **3**, 2048 × 512 real points (F_2, F_1) in using States-TPPI for phase sensitivity in the indirectly detected dimension were used. A spectral width of 10 ppm × 10 ppm centered around 5 ppm was covered. Tw o scans (ns) were acquired per point with 200 ms acquisition time and 6 s interscan delay (d1). The spectrum was processed to 4096 × 4096 hypercomplex points with a cos2 window function. Taking advantage of not being limited by the size of the FID (td) in F2, for taxol (**4**) the experiment was acquired with 20000 × 640 real points (F_2, F_1), leading to an acquisition time (aq) of 2 s and providing spectral resolution in F_2 of 0.5 Hz. The interscan delay (d1) was set to 4 s in order to have total recycling delay of 6 s to maintain the quantitative conditions.
The assignments of ^{1}H NMR signals for **1** was based on previous work,[27] as well as for **3**[37] and for **4**.[41]
The lists of integrals are provided in a file together with the MATLAB source code.

Structure generation

Configurations were generated using LigPrep in Schrödinger Maestro. Conformational searches and redundant conformation elimination were performed with the Schrödinger MacroModel suite using the MMFFs force field for $CDCl_3$ as solvent and an energy cutoff of 3kcal/mol. [https://www.schrodinger.com]

Note on Matlab source code

The Matlab script that was written for this work is provided in an external compressed file which also includes xyz structure files and the list of experimental NOE integrals. It is released under Creative Commons Attribution-ShareAlike 4.0 International (CC BY-SA 4.0) license as defined by https://creativecommons.org/licenses/by-sa/4.0/legalcode.

Fit results for strychnine configuration selection

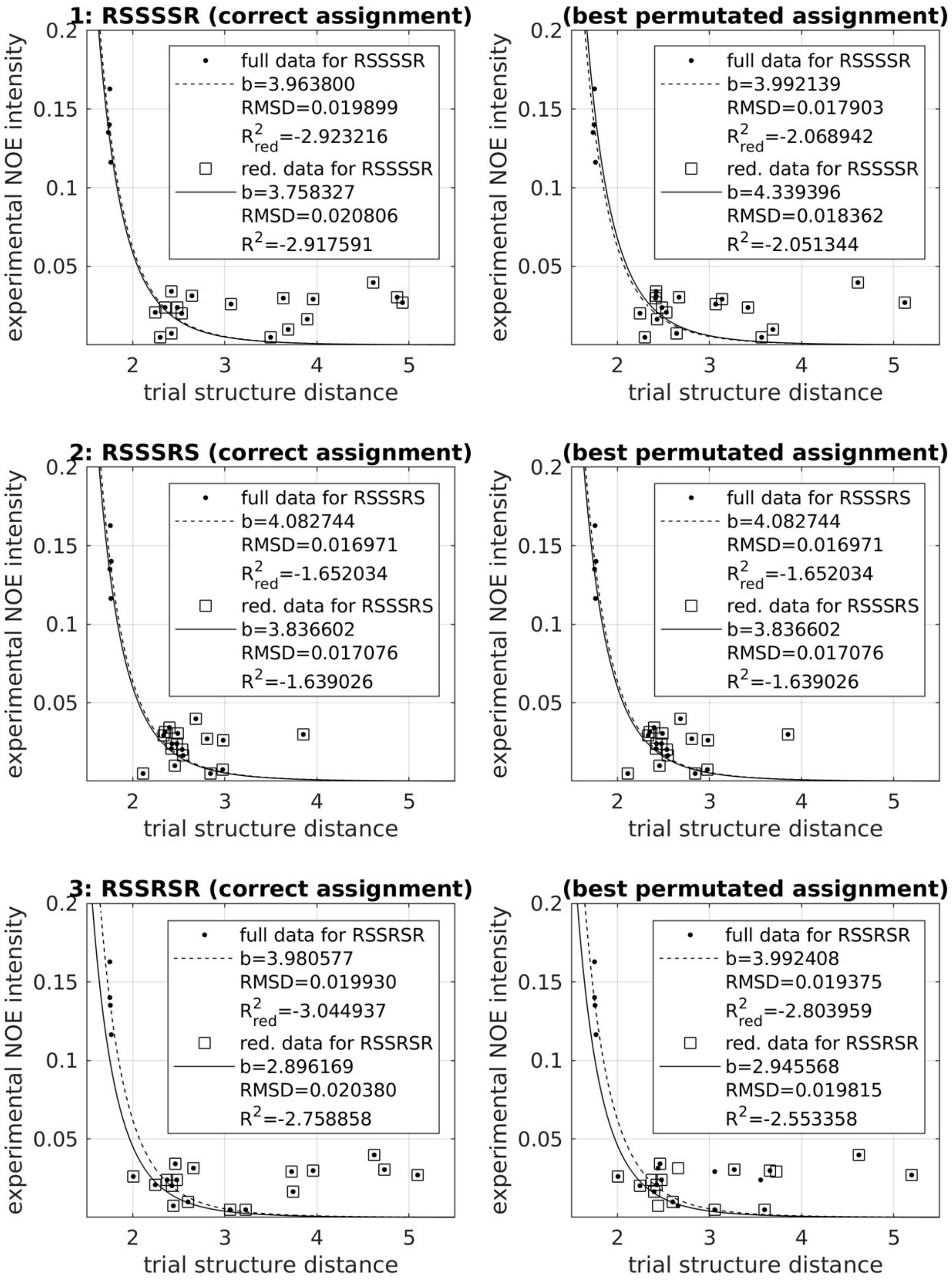

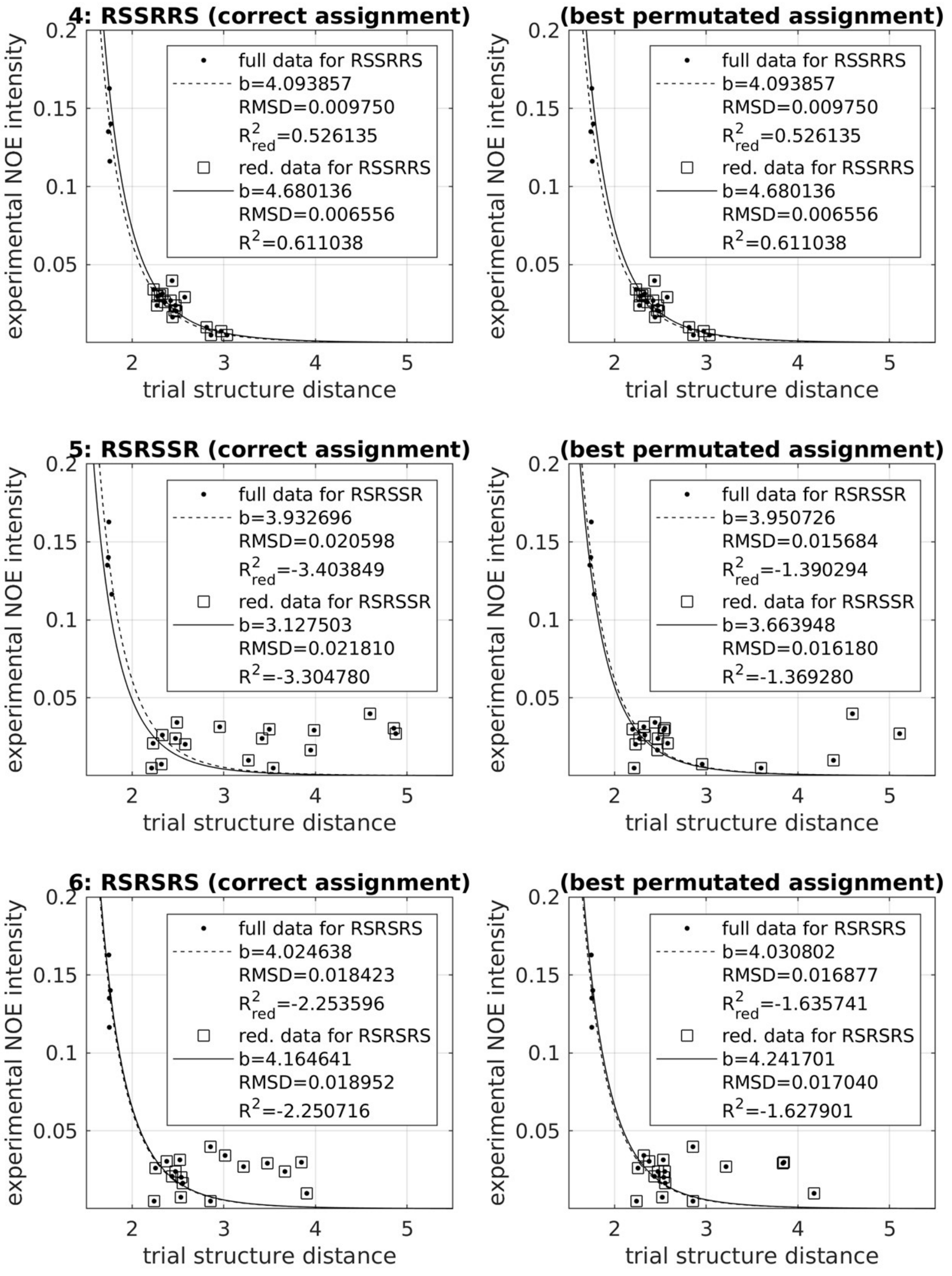

4: RSSRRS (correct assignment)
(best permutated assignment)
full data for RSSRRS
b=4.093857
RMSD=0.009750
R^2_{red}=0.526135
red. data for RSSRRS
b=4.680136
RMSD=0.006556
R^2=0.611038
5: RSRSSR (correct assignment)
(best permutated assignment)
full data for RSRSSR
b=3.932696
RMSD=0.020598
R^2_{red}=-3.403849
red. data for RSRSSR
b=3.127503
RMSD=0.021810
R^2=-3.304780
full data for RSRSSR
b=3.950726
RMSD=0.015684
R^2_{red}=-1.390294
red. data for RSRSSR
b=3.663948
RMSD=0.016180
R^2=-1.369280
6: RSRSRS (correct assignment)
(best permutated assignment)
full data for RSRSRS
b=4.024638
RMSD=0.018423
R^2_{red}=-2.253596
red. data for RSRSRS
b=4.164641
RMSD=0.018952
R^2=-2.250716
full data for RSRSRS
b=4.030802
RMSD=0.016877
R^2_{red}=-1.635741
red. data for RSRSRS
b=4.241701
RMSD=0.017040
R^2=-1.627901
experimental NOE intensity
trial structure distance

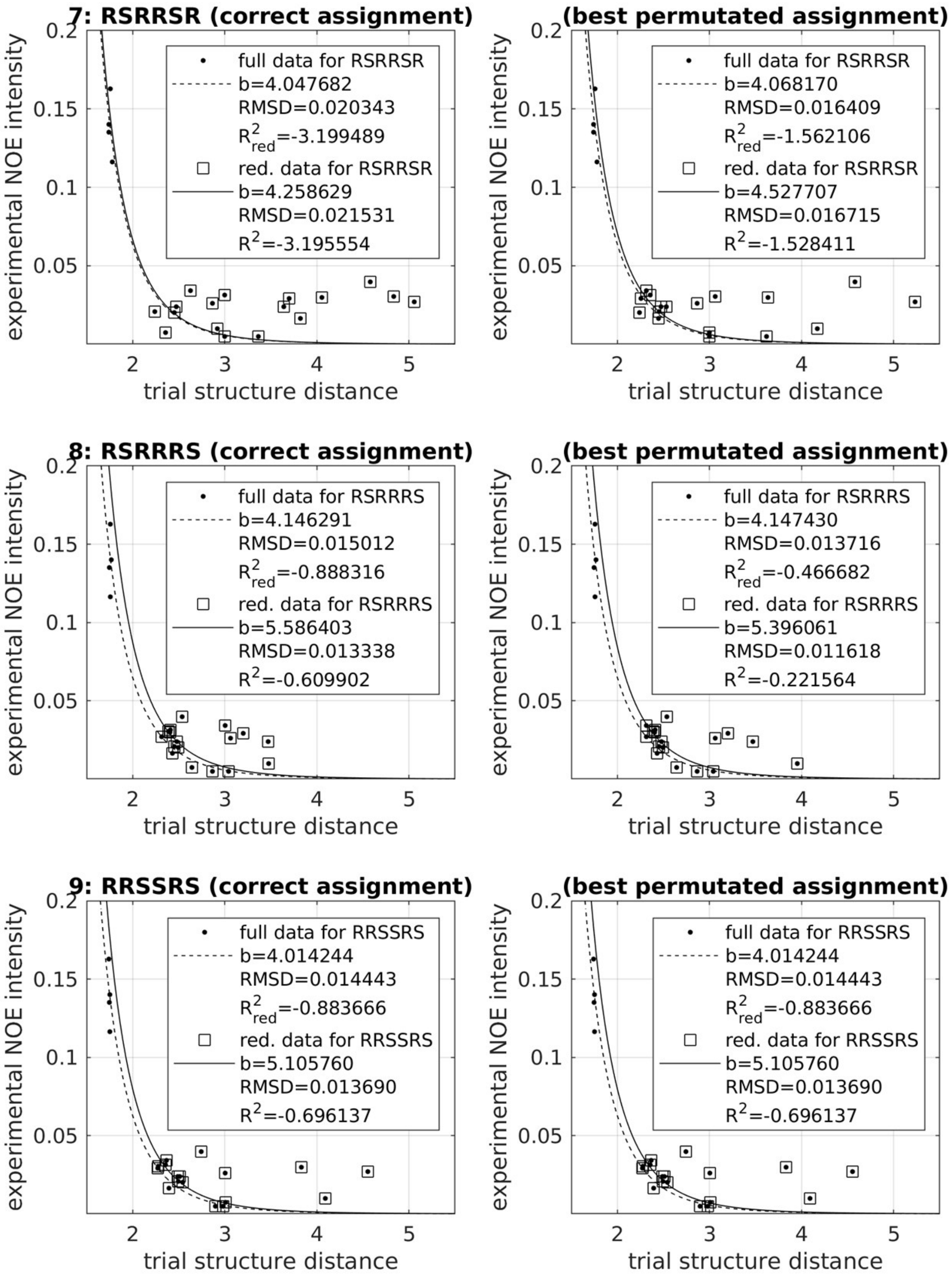
7: RSRRSR (correct assignment)
full data for RSRRSR
b=4.047682
RMSD=0.020343
R2red=-3.199489
red. data for RSRRSR
b=4.258629
RMSD=0.021531
R2=-3.195554
(best permutated assignment)
full data for RSRRSR
b=4.068170
RMSD=0.016409
R2red=-1.562106
red. data for RSRRSR
b=4.527707
RMSD=0.016715
R2=-1.528411
8: RSRRRS (correct assignment)
full data for RSRRRS
b=4.146291
RMSD=0.015012
R2red=-0.888316
red. data for RSRRRS
b=5.586403
RMSD=0.013338
R2=-0.609902
(best permutated assignment)
full data for RSRRRS
b=4.147430
RMSD=0.013716
R2red=-0.466682
red. data for RSRRRS
b=5.396061
RMSD=0.011618
R2=-0.221564
9: RRSSRS (correct assignment)
full data for RRSSRS
b=4.014244
RMSD=0.014443
R2red=-0.883666
red. data for RRSSRS
b=5.105760
RMSD=0.013690
R2=-0.696137
(best permutated assignment)
full data for RRSSRS
b=4.014244
RMSD=0.014443
R2red=-0.883666
red. data for RRSSRS
b=5.105760
RMSD=0.013690
R2=-0.696137
experimental NOE intensity
trial structure distance

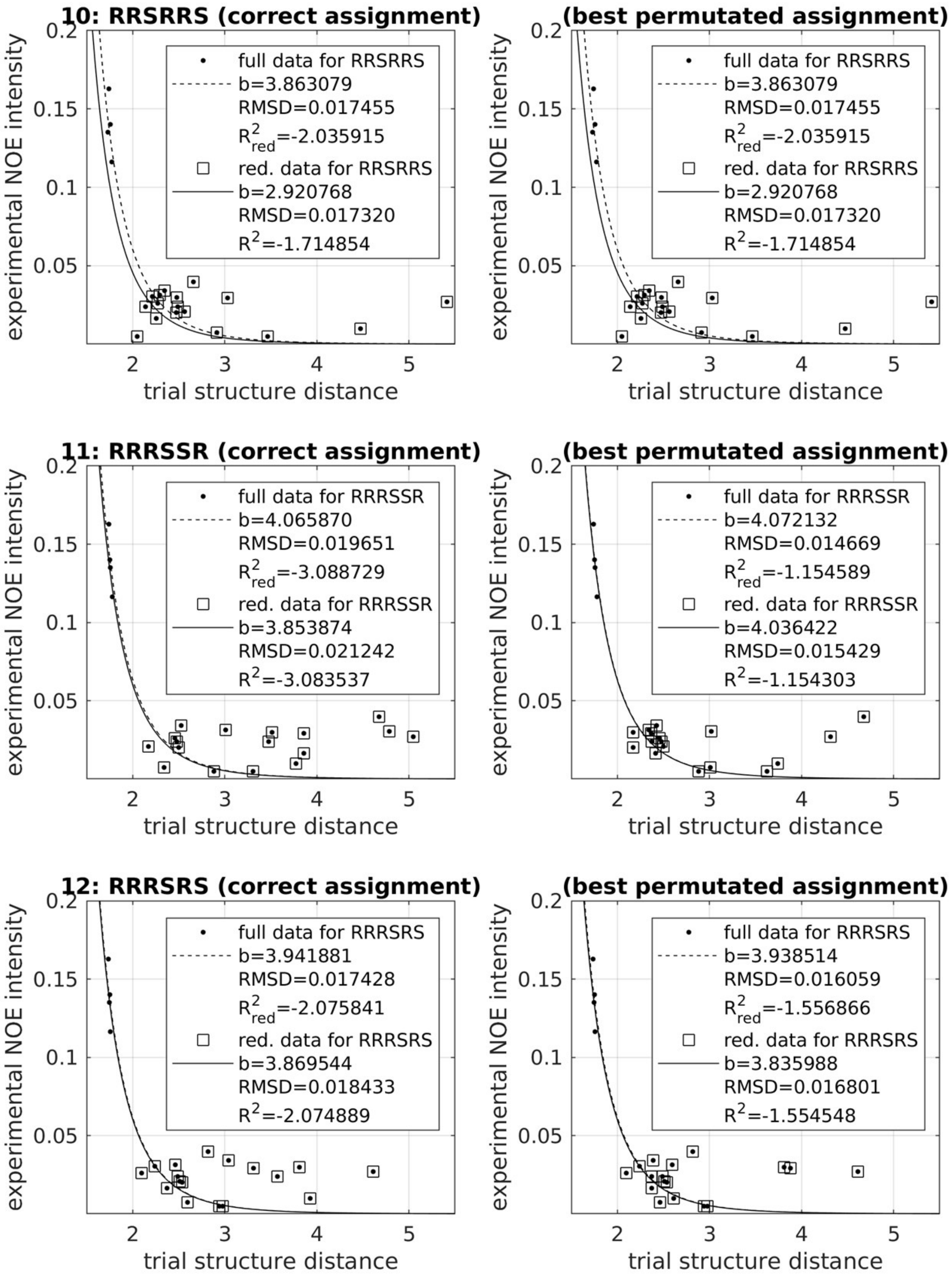
10: RRSRRS (correct assignment)
(best permutated assignment)
experimental NOE intensity
trial structure distance
full data for RRSRRS
b=3.863079
RMSD=0.017455
R2red=-2.035915
red. data for RRSRRS
b=2.920768
RMSD=0.017320
R2=-1.714854
full data for RRSRRS
b=3.863079
RMSD=0.017455
R2red=-2.035915
red. data for RRSRRS
b=2.920768
RMSD=0.017320
R2=-1.714854
11: RRRSSR (correct assignment)
(best permutated assignment)
full data for RRRSSR
b=4.065870
RMSD=0.019651
R2red=-3.088729
red. data for RRRSSR
b=3.853874
RMSD=0.021242
R2=-3.083537
full data for RRRSSR
b=4.072132
RMSD=0.014669
R2red=-1.154589
red. data for RRRSSR
b=4.036422
RMSD=0.015429
R2=-1.154303
12: RRRSRS (correct assignment)
(best permutated assignment)
full data for RRRSRS
b=3.941881
RMSD=0.017428
R2red=-2.075841
red. data for RRRSRS
b=3.869544
RMSD=0.018433
R2=-2.074889
full data for RRRSRS
b=3.938514
RMSD=0.016059
R2red=-1.556866
red. data for RRRSRS
b=3.835988
RMSD=0.016801
R2=-1.554548

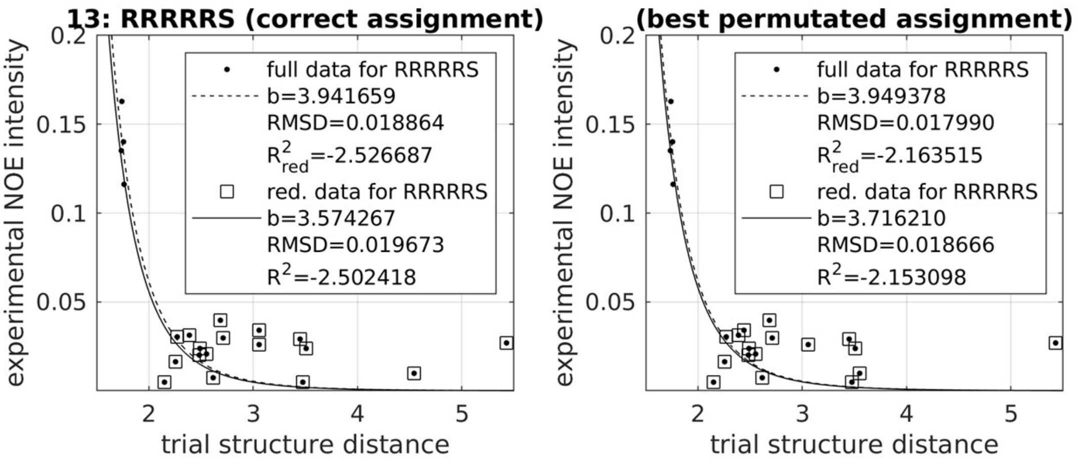

Figure S1. Comparison of experimental and calculated data; result of fitting procedure for all isomers. R_{red} ^{2} corresponds to R^2 of data without intra-CH_2 integrals using the full data for the fitting procedure. Reduced data ("red. data") corresponds to data without intra-CH_2 integrals altogether. On the left side, correct assignment is used; on the right side, assignment of diastereotopic CH_2 groups is permutated for each isomer individually to obtain the best fit.

Additional quantitative strychnine scoring data

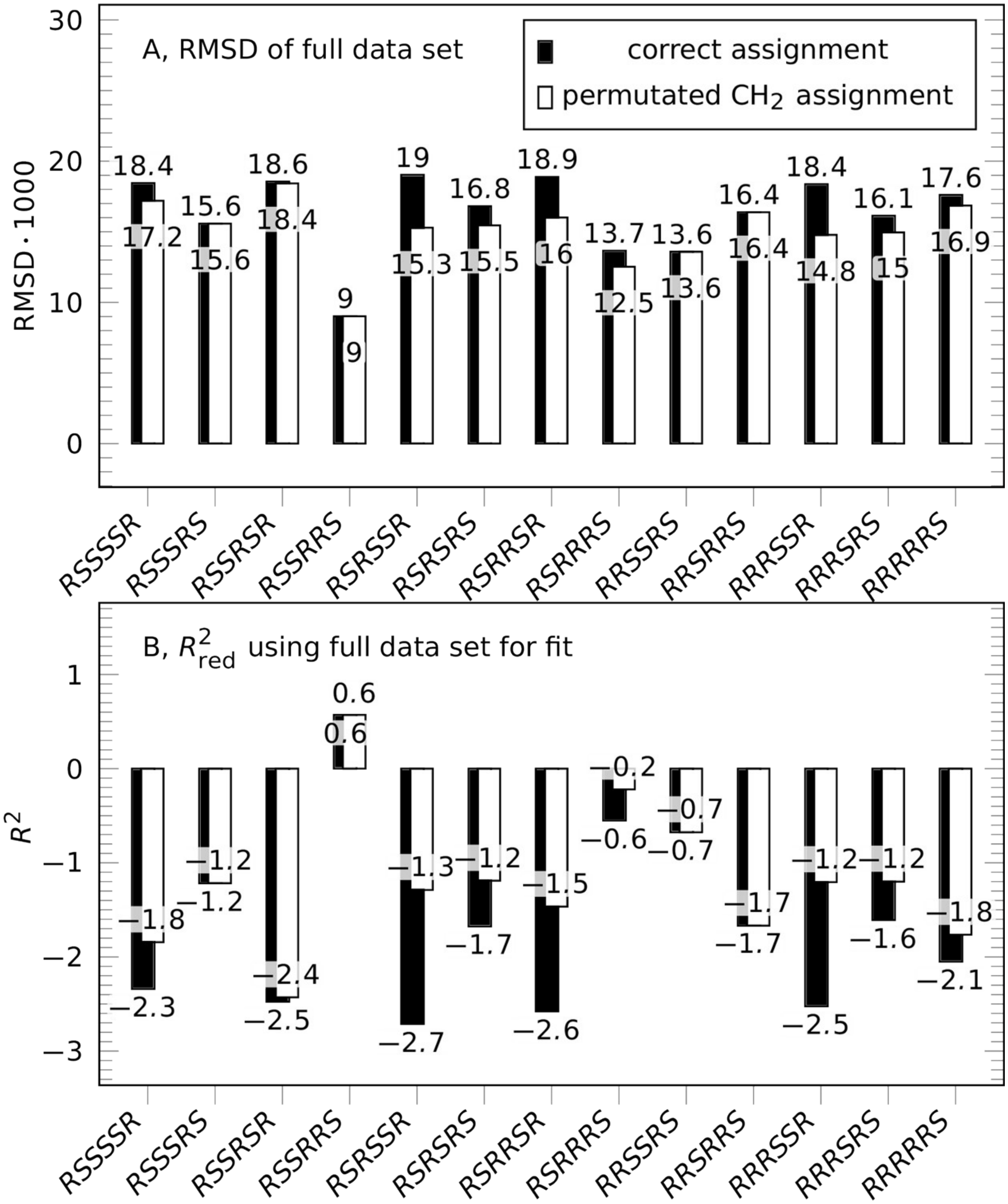

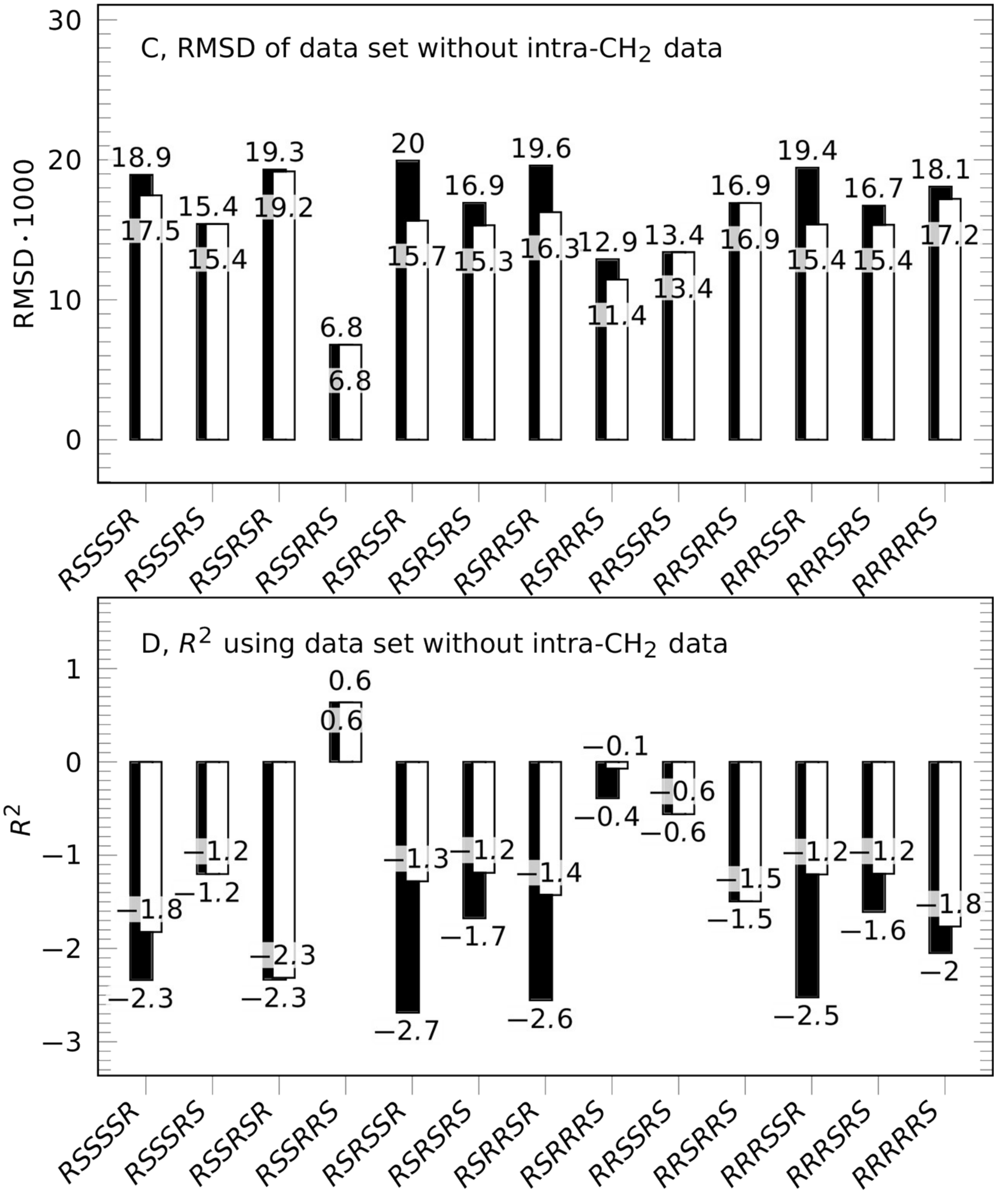

Figure S2. Result of structure selection using different data sets and scoring functions. In (A) and (B), all data (including intra-CH_2 data) is used for the fit. R_{red}^2 in (B) corresponds to R_2 scoring of data without intra-CH_2 integrals to the fit results from the full data, while for (C) and (D) the intra-CH2 integrals were removed from the data for the fitting procedure as well as for the scoring.

Fit results for artemisinin configuration selection

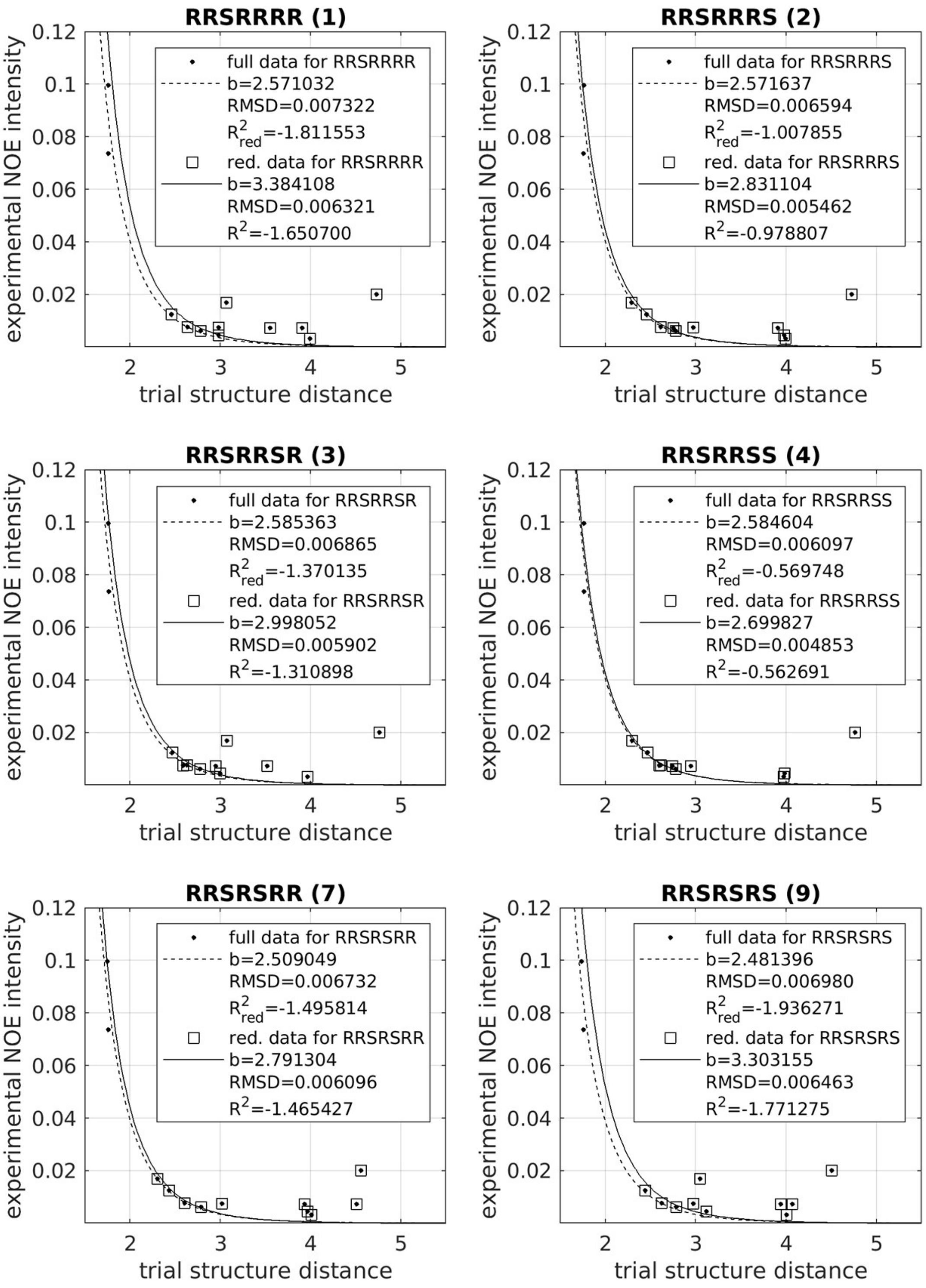

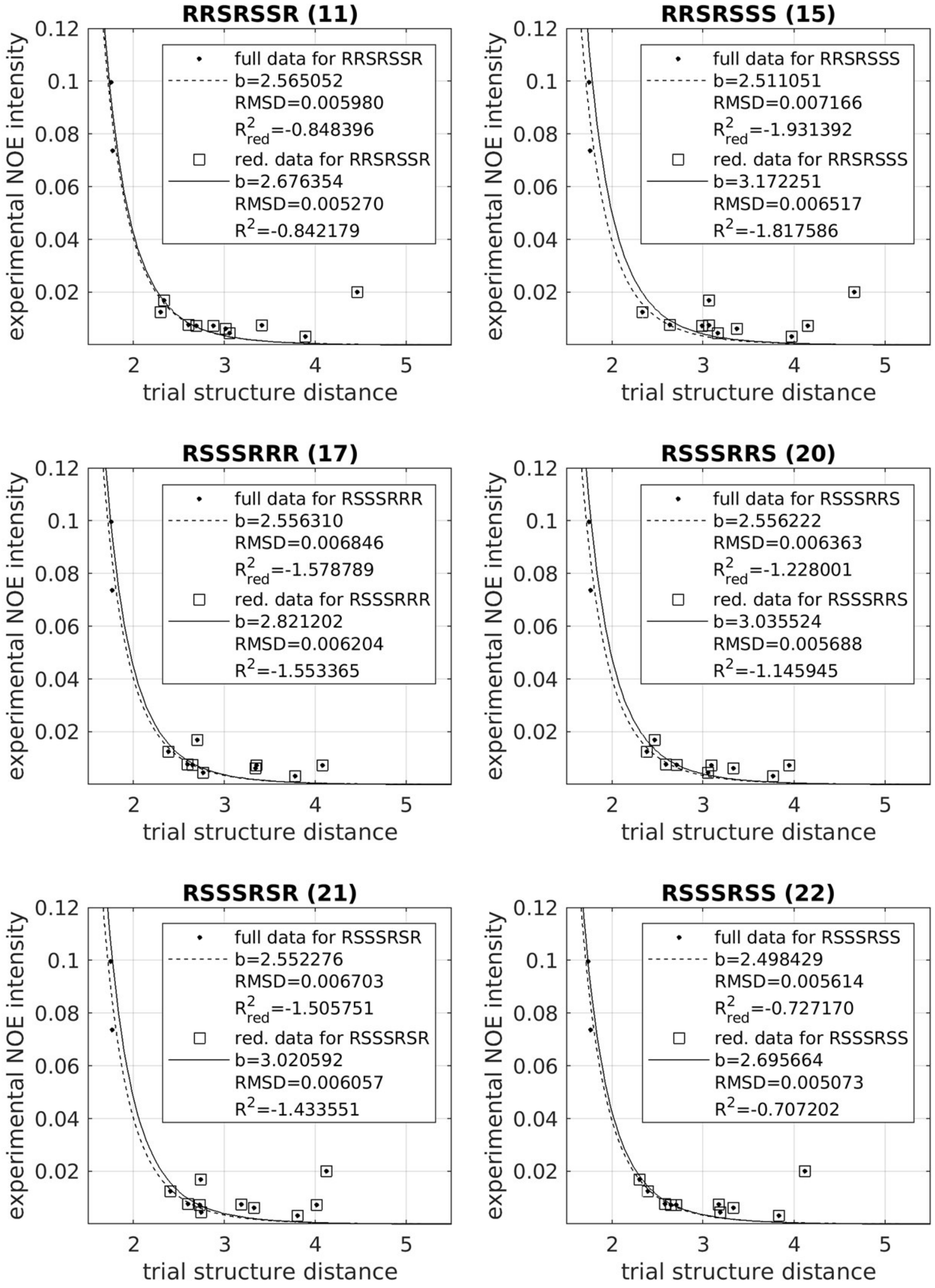
RRSRSSR (11)
full data for RRSRSSR
b=2.565052
RMSD=0.005980
R^2_{red}=-0.848396
red. data for RRSRSSR
b=2.676354
RMSD=0.005270
R^2=-0.842179
RRSRSSS (15)
full data for RRSRSSS
b=2.511051
RMSD=0.007166
R^2_{red}=-1.931392
red. data for RRSRSSS
b=3.172251
RMSD=0.006517
R^2=-1.817586
RSSSRRR (17)
full data for RSSSRRR
b=2.556310
RMSD=0.006846
R^2_{red}=-1.578789
red. data for RSSSRRR
b=2.821202
RMSD=0.006204
R^2=-1.553365
RSSSRRS (20)
full data for RSSSRRS
b=2.556222
RMSD=0.006363
R^2_{red}=-1.228001
red. data for RSSSRRS
b=3.035524
RMSD=0.005688
R^2=-1.145945
RSSSRSR (21)
full data for RSSSRSR
b=2.552276
RMSD=0.006703
R^2_{red}=-1.505751
red. data for RSSSRSR
b=3.020592
RMSD=0.006057
R^2=-1.433551
RSSSRSS (22)
full data for RSSSRSS
b=2.498429
RMSD=0.005614
R^2_{red}=-0.727170
red. data for RSSSRSS
b=2.695664
RMSD=0.005073
R^2=-0.707202
experimental NOE intensity
trial structure distance
0.12
0.1
0.08
0.06
0.04
0.02
2
3
4
5

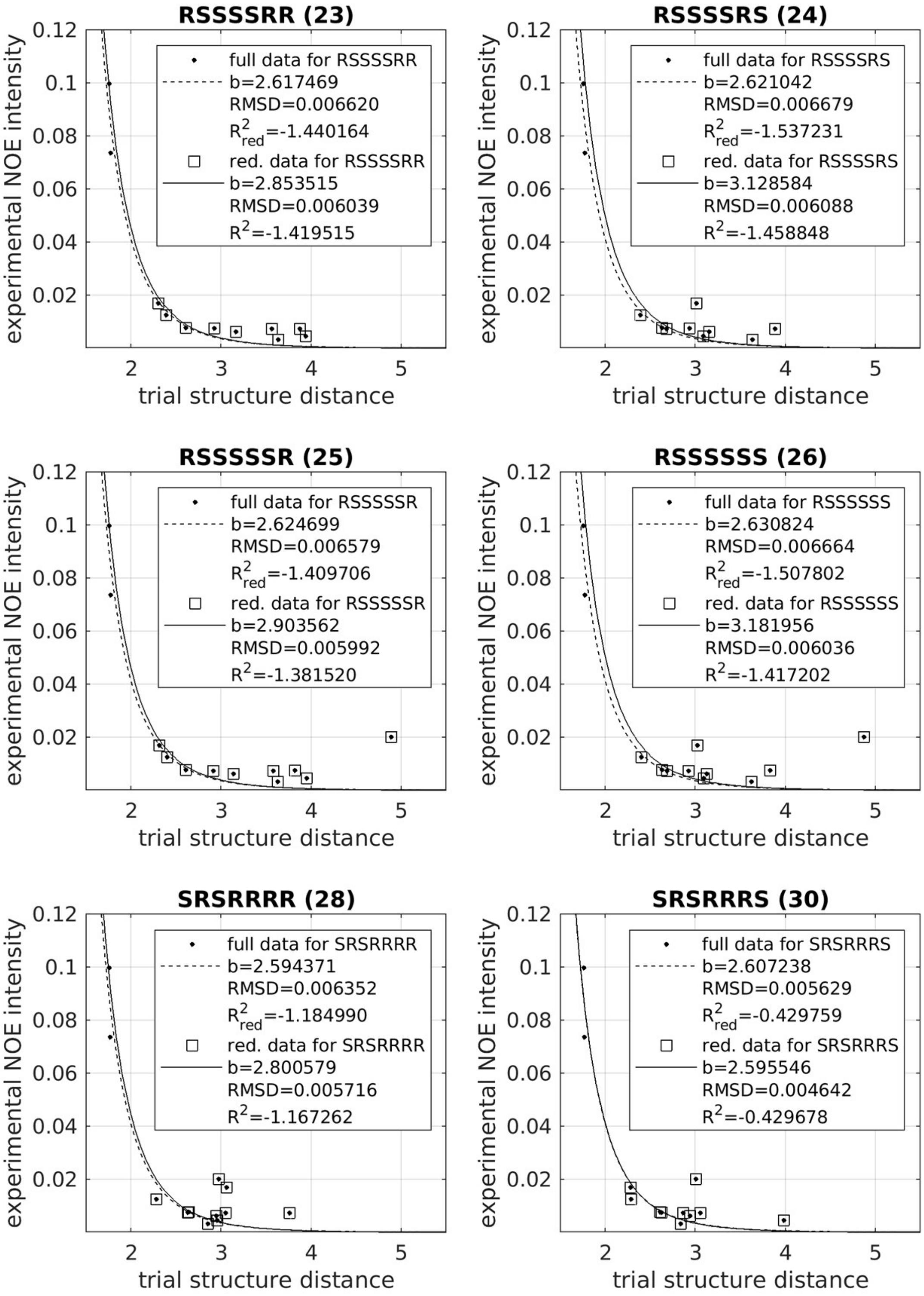
RSSSSRR (23)
experimental NOE intensity
trial structure distance
full data for RSSSSRR
b=2.617469
RMSD=0.006620
R^2_{red}=-1.440164
red. data for RSSSSRR
b=2.853515
RMSD=0.006039
R^2=-1.419515
RSSSSRS (24)
full data for RSSSSRS
b=2.621042
RMSD=0.006679
R^2_{red}=-1.537231
red. data for RSSSSRS
b=3.128584
RMSD=0.006088
R^2=-1.458848
RSSSSSR (25)
full data for RSSSSSR
b=2.624699
RMSD=0.006579
R^2_{red}=-1.409706
red. data for RSSSSSR
b=2.903562
RMSD=0.005992
R^2=-1.381520
RSSSSSS (26)
full data for RSSSSSS
b=2.630824
RMSD=0.006664
R^2_{red}=-1.507802
red. data for RSSSSSS
b=3.181956
RMSD=0.006036
R^2=-1.417202
SRSRRRR (28)
full data for SRSRRRR
b=2.594371
RMSD=0.006352
R^2_{red}=-1.184990
red. data for SRSRRRR
b=2.800579
RMSD=0.005716
R^2=-1.167262
SRSRRRS (30)
full data for SRSRRRS
b=2.607238
RMSD=0.005629
R^2_{red}=-0.429759
red. data for SRSRRRS
b=2.595546
RMSD=0.004642
R^2=-0.429678

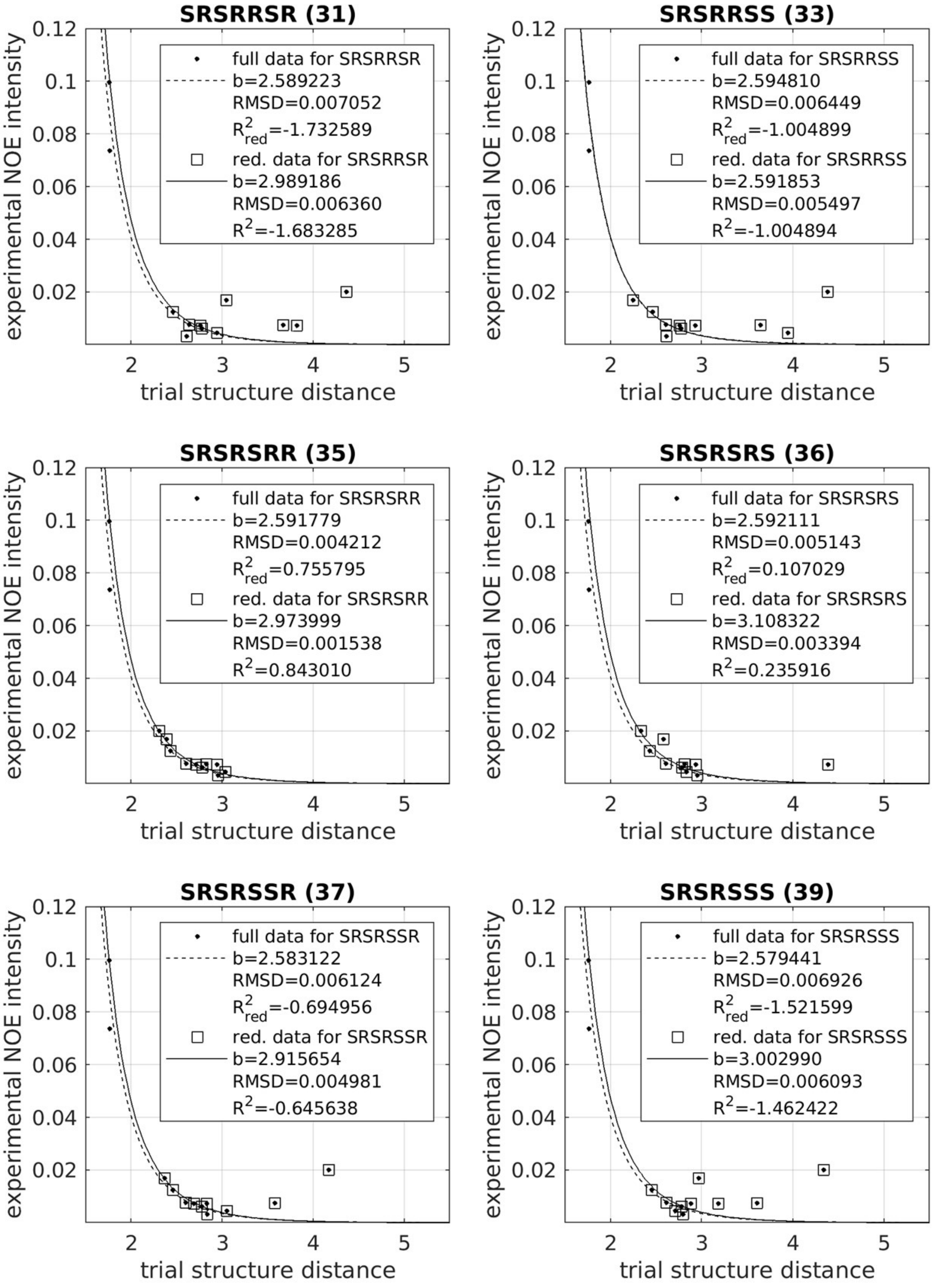
SRSRRSR (31)
full data for SRSRRSR
b=2.589223
RMSD=0.007052
R2red=-1.732589
red. data for SRSRRSR
b=2.989186
RMSD=0.006360
R2=-1.683285
SRSRRSS (33)
full data for SRSRRSS
b=2.594810
RMSD=0.006449
R2red=-1.004899
red. data for SRSRRSS
b=2.591853
RMSD=0.005497
R2=-1.004894
SRSRSRR (35)
full data for SRSRSRR
b=2.591779
RMSD=0.004212
R2red=0.755795
red. data for SRSRSRR
b=2.973999
RMSD=0.001538
R2=0.843010
SRSRSRS (36)
full data for SRSRSRS
b=2.592111
RMSD=0.005143
R2red=0.107029
red. data for SRSRSRS
b=3.108322
RMSD=0.003394
R2=0.235916
SRSRSSR (37)
full data for SRSRSSR
b=2.583122
RMSD=0.006124
R2red=-0.694956
red. data for SRSRSSR
b=2.915654
RMSD=0.004981
R2=-0.645638
SRSRSSS (39)
full data for SRSRSSS
b=2.579441
RMSD=0.006926
R2red=-1.521599
red. data for SRSRSSS
b=3.002990
RMSD=0.006093
R2=-1.462422
experimental NOE intensity
trial structure distance
0.12
0.1
0.08
0.06
0.04
0.02
2
3
4
5

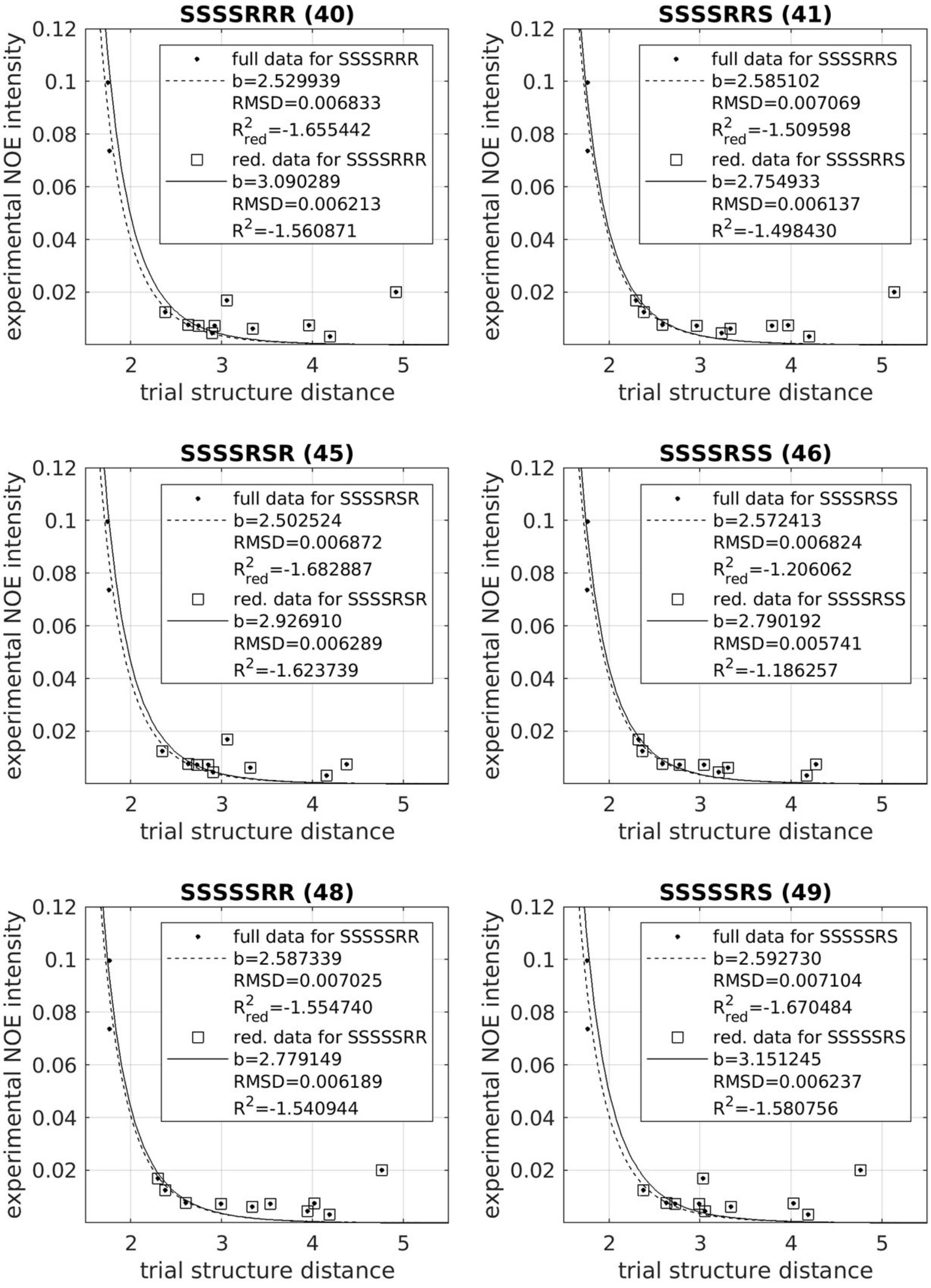
SSSSRRR (40)
full data for SSSSRRR
b=2.529939
RMSD=0.006833
R2red=-1.655442
red. data for SSSSRRR
b=3.090289
RMSD=0.006213
R2=-1.560871
SSSSRRS (41)
full data for SSSSRRS
b=2.585102
RMSD=0.007069
R2red=-1.509598
red. data for SSSSRRS
b=2.754933
RMSD=0.006137
R2=-1.498430
SSSSRSR (45)
full data for SSSSRSR
b=2.502524
RMSD=0.006872
R2red=-1.682887
red. data for SSSSRSR
b=2.926910
RMSD=0.006289
R2=-1.623739
SSSSRSS (46)
full data for SSSSRSS
b=2.572413
RMSD=0.006824
R2red=-1.206062
red. data for SSSSRSS
b=2.790192
RMSD=0.005741
R2=-1.186257
SSSSSRR (48)
full data for SSSSSRR
b=2.587339
RMSD=0.007025
R2red=-1.554740
red. data for SSSSSRR
b=2.779149
RMSD=0.006189
R2=-1.540944
SSSSSRS (49)
full data for SSSSSRS
b=2.592730
RMSD=0.007104
R2red=-1.670484
red. data for SSSSSRS
b=3.151245
RMSD=0.006237
R2=-1.580756
experimental NOE intensity
trial structure distance

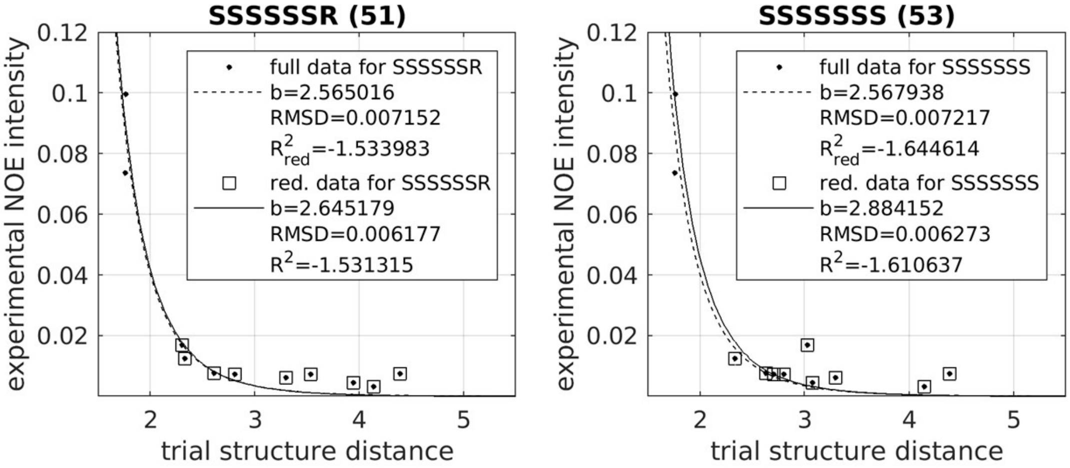

Figure S3. Comparison of experimental and calculated data for artemisinin; result of fitting procedure for the best conformer of each configuration. $R_{red}{}^2$ corresponds to R^2 of data without intra-CH_2 integrals using the full data for the fitting procedure. Reduced data (“red. data") corresponds to data without intra-CH_2 integrals altogether.

Fit results for taxol configuration selection

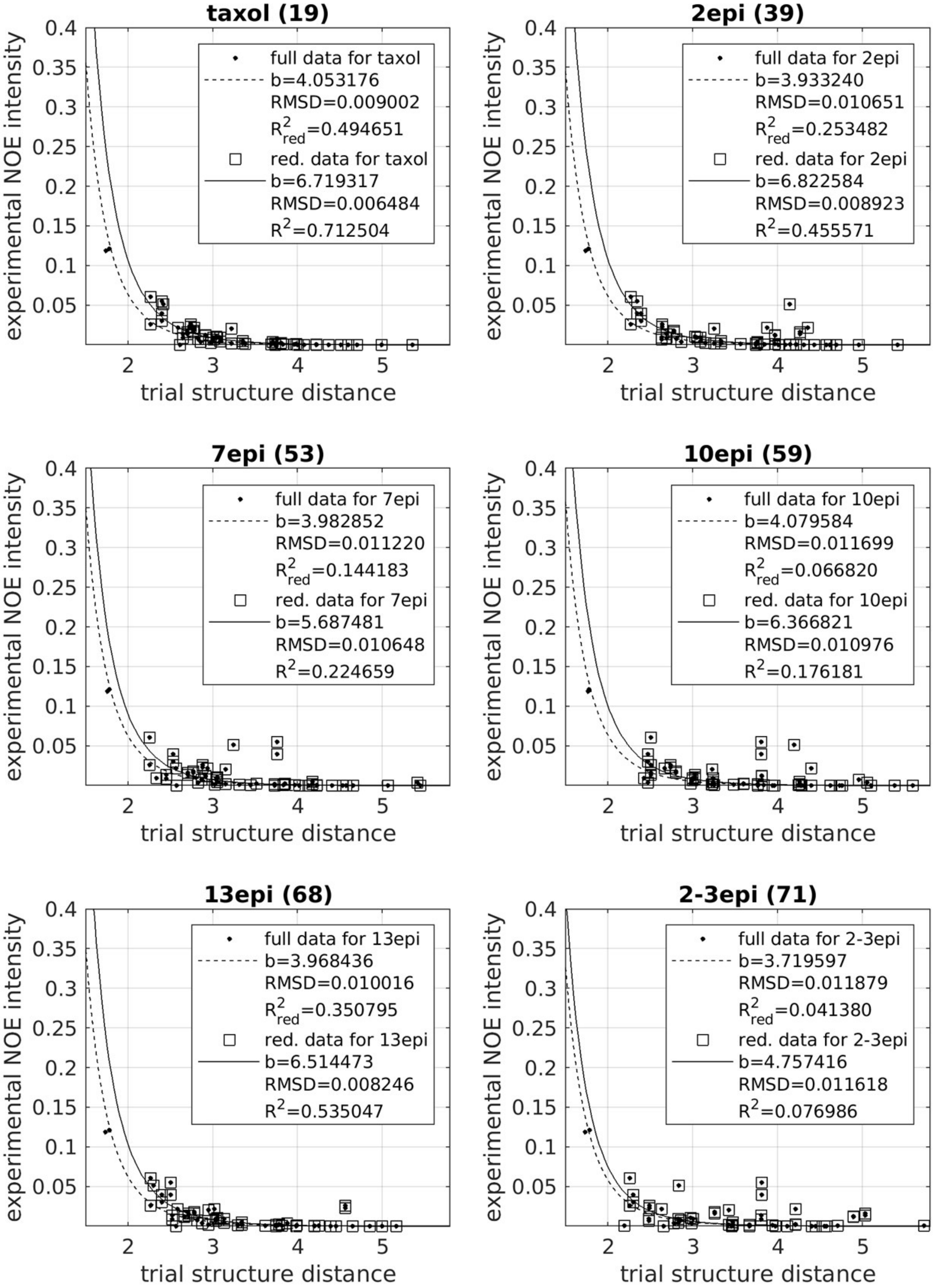

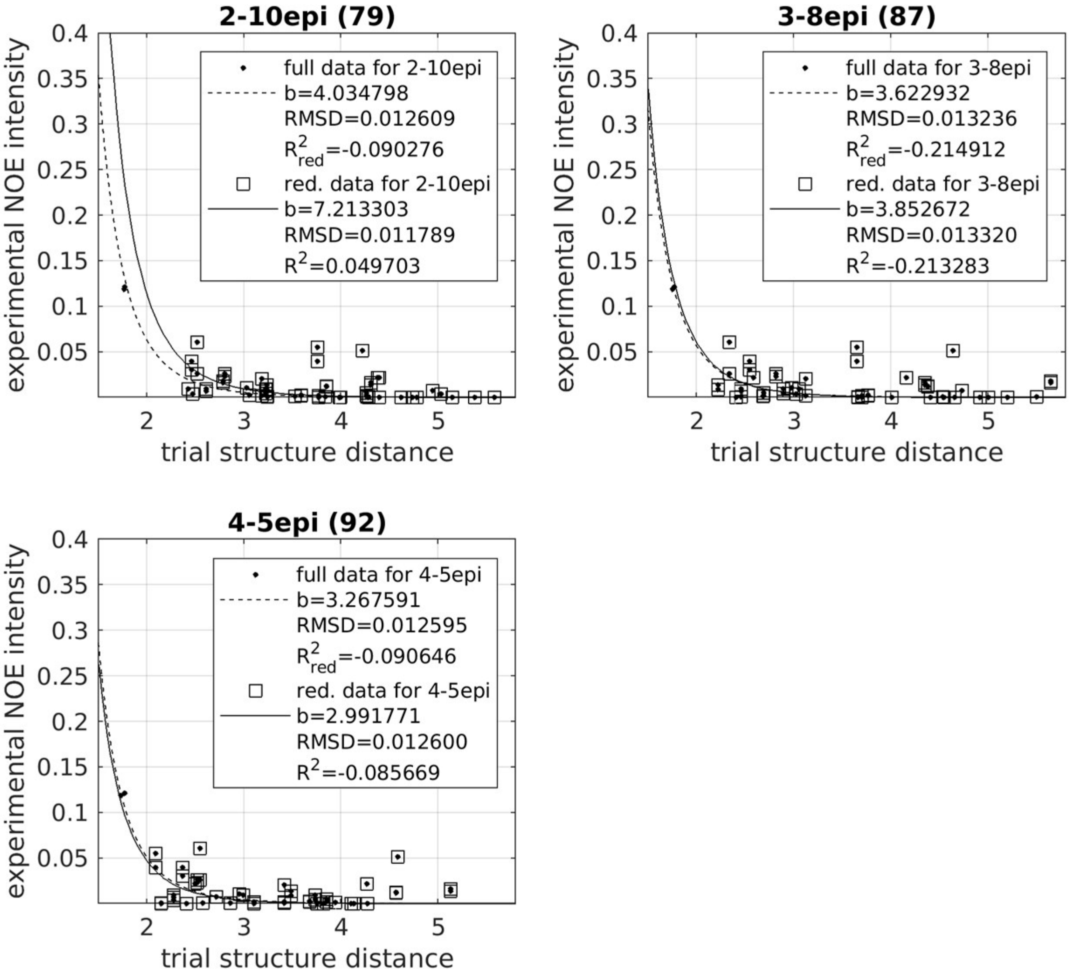

Figure S4. Comparison of experimental and calculated data for taxol; result of fitting procedure for the best conformer of each configuration. R_{red} ^{2} corresponds to R^2 of data without intra-CH_2 integrals using the full data for the fitting procedure. Reduced data (“red. data") corresponds to data without intra-CH_2 integrals altogether.
